# Simple obstructive sleep apnea patients without hypertension or diabetes accelerate kidney dysfunction: a population follow-up cohort study from Taiwan

**DOI:** 10.1007/s11325-016-1376-2

**Published:** 2016-07-05

**Authors:** Yu-Sheng Lin, Pi-Hua Liu, Shi-Wei Lin, Li-Pang Chuang, Wan-Jing Ho, Yu-Ting Chou, Kuo-Chang Juan, Min-Tzu Lo, Pao-Hsien Chu, Ning-Hung Chen

**Affiliations:** 1 Department of Cardiology, Chang Gung Memorial Hospital and Chang Gung University, Taipei, Taiwan; 2 Healthcare Center, Chang Gung Memorial Hospital and Chang Gung University, Taipei, Taiwan; 3 Clinical Informatics and Medical Statistics Research Center, College of Medicine, Chang Gung University, Taoyuan, Taiwan; 4 Sleep Center, Department of Pulmonary and Critical Care Medicine, Chang Gung Memorial Hospital, No. 123, Dinghu RD., Guishan Township, Taoyuan County, Taiwan Republic of China; 5 Graduate Institute of Clinical Medical Sciences, College of Medicine, Chang Gung University, Taoyuan, Taiwan; 6 Department of Nephrology, Chang Gung Memorial Hospital, Taipei, Taiwan; 7 Department of Radiology, University of California, San Diego, USA; 8 Heart Failure Center, Chang Gung Memorial Hospital and Chang Gung University, Taipei, Taiwan; 9 Department of Internal Medicine, Chang Gung Memorial Hospital, Taoyuan, Taiwan

**Keywords:** Obstructive sleep apnea, Kidney dysfunction, Diabetes, Hypertension

## Abstract

**Backgrounds:**

Obstructive sleep apnea (OSA) is common in patients on hemodialysis, but its correlation with chronic kidney disease (CKD) is not clear. We aimed to clarify the relationship between OSA without hypertension or diabetes and incidence of CKD in Taiwan.

**Methods:**

This population-based cohort study consisted of patients with newly diagnosed OSA between 2000 and 2009. The comparison cohort was matched for age, sex, diabetes mellitus, and hypertension. All subjects previously diagnosed with acute or chronic kidney disease were excluded. The primary end point was newly diagnosed CKD.

**Results:**

We identified 6866 subjects with OSA during the 10-year study period. The median duration until development of CKD in the OSA cohort was 3.2 years, 2.5 months earlier than that in the non-OSA cohort. After exclusion of hypertension and diabetes, 4319 OSA patients was identified and the hazard ratio (HR) of CKD with OSA was 1.37 (95 % confidence interval [CI], 1.05–1.77; *p* = 0.019). In the subgroup analysis, an increased incidence of CKD in OSA was observed in women (HR, 1.41; 95 % CI, 1.12–1.78; *p* = 0.0036).

**Conclusions:**

This longitudinal population-based cohort study provides evidence that patients with OSA even without diabetes or hypertension are at higher risk of developing CKD over the next 3 years and nearly 2.5 months earlier than the non-OSA cohort, particularly women.

## Introduction

Obstructive sleep apnea (OSA) is a disorder that affects 2–4 % of adults in the general population of Taiwan and worldwide [1, 2]. OSA patients increase hypertension and diabetes mellitus risk [3–6]. Cardiovascular and neurocognitive complications are the most common and severe consequences noted in these patients [7]. The underlying mechanisms are complex and involve oxidative stress and inflammation, sympathetic tone stimulation, and fluid shifting, which may negatively affect kidney function; thus, it is not surprising that OSA is highly prevalent among patients on hemodialysis [8–10].

Chronic kidney disease (CKD) affects 10–13 % of the general population in Taiwan and is associated with substantial morbidity and mortality rates, particularly with regard to cardiovascular diseases and end-stage kidney disease [11]. Diabetes and hypertension are the two major risk factors for CKD [12–14]. Our previous study found that patients with OSA without diabetes and hypertension have a higher prevalence of impaired renal function [15]. Sim et al. reported an association of OSA and CKD in cross-sectional study and Ahmed et al, reported that nocturnal hypoxia may have a higher prevalence for loss of kidney function [16–18]. TAHRANI reported a small diabetes cohort that higher prevalence of OSA will enhance the declaration of eGFR in 2-year follow up [19]. Molnar reported higher incidence of CKD associated to OSA veterans in a 3 million USA cohort subgroup analysis [20]. Recently, Lee reported OSA patients predisposing to the development of CKD and end-stage renal disease (ESRD) [21]. However, the interaction between CKD and pure OSA without hypertension or diabetes is not confirmed due to selection bias. To clarify the relationship of OSA and CKD, we aimed to design this retrospective longitudinal population-based case-control cohort study especially focused on pure OSA without diabetes or hypertension.

## Materials and methods

### Data source

This was a retrospective cohort study that used the Longitudinal Health Insurance Database 2000 (LHID2000) in Taiwan, a subset of the National Health Insurance Research Database (NHIRD) (http://nhird.nhri.org.tw/date_01.html), containing all original claims data for one million randomly sampled insured patients in 2000. The NHIRD comprises medical and pharmacy claims including inpatient and outpatient diagnoses and procedures based on the International Classification of Diseases, 9th Revision, Clinical Modification (ICD-9-CM). The data are considered reasonably representative of Taiwan residents, since >99 % of Taiwan’s population was enrolled in the NHI program [22].

All personal identifiers were encrypted by the Bureau of NHI prior to being released to researchers. The institutional review board of Chang Gung Memorial Hospital approved this retrospective study (IRB number 101-5057B) and waived the requirement for informed consent.

### Study and comparison cohort

The study cohort consisted of 6866 adult patients (aged ≥18 years) with newly diagnosed OSA between 2000 and 2009. A diagnosis of OSA included sleep apnea with hypersomnia (ICD-9-CM 780.53), sleep apnea with insomnia (ICD-9-CM 780.51), and sleep apnea unspecified (ICD-9-CM 780.57) as previously reported [23]. Due to Taiwan’s medical coverage system, there must be a diagnosis for further disease checkup in clinic; we only enrolled patients with OSA diagnosis completing three or more outpatient verification visits within 1 year or those who had been hospitalized with a diagnosis of OSA. The index date for each patient was that of the first OSA diagnosis.

Acute or chronic kidney disease diagnosis is based on the ICD-9-CM code including 581 (nephritic syndrome), 582 (chronic glomerulonephritis), 583 (nephritis and nephropathy not specified as acute or chronic), 584 (acute kidney failure), and 585 (chronic kidney disease) OSA patients, and patients who were previously diagnosed with acute or CKD (ICD-9-CM 581-585) before the index date were excluded from the analyses.

**Fig. 1:**
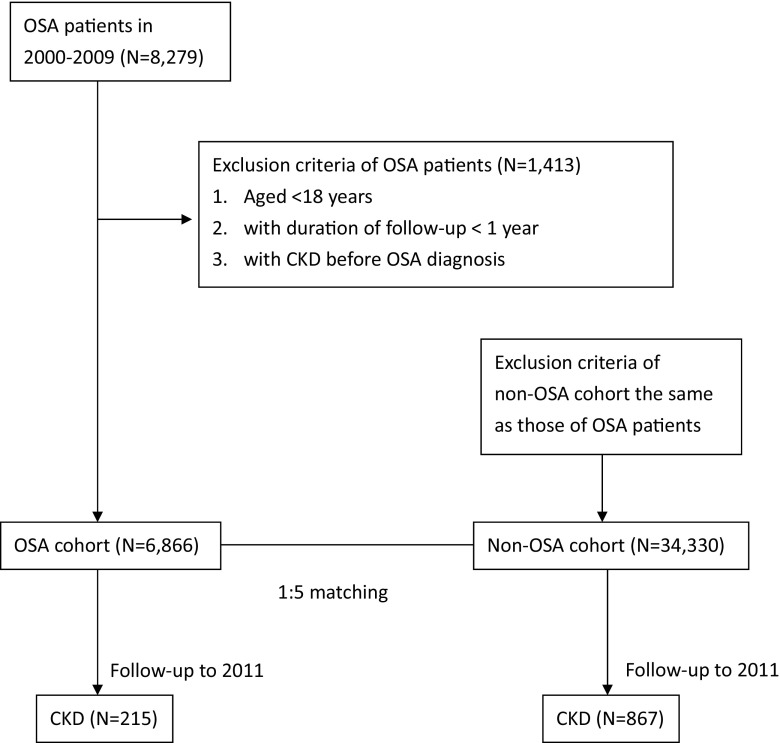
Flow chart of ascertainment of OSA and non-OSA cohort

The comparison cohort consisted of adults (age ≥18 years) without OSA (non-OSA) who were randomly selected from the remaining individuals after excluding any diagnosis with OSA in the LHID2000 database. All non-OSA subjects were matched with the study cohort for age, sex, diabetes (ICD-9-CM 250), hypertension (ICD-9-CM 401.0, 401.1, and 401.9), and index year. None of the non-OSA subjects had been previously diagnosed with acute or CKD (ICD-9-CM 581-585) before the index year. All study and comparison cohort members were followed up for at least 1 year. Details are described on the right of the flow chart (Fig. 1, right side) and Table 1.

**Table 1:** Characteristic of matching variables in OSA and non-OSA cohorts at baseline of follow-up

	OSA (%)	Non-OSA (%)
	(*N* = 6866)	(*N* = 34,330)
Age at baseline		
18–29	949 (13.82)	4745 (13.82)
30–39	1460 (21.26)	7300 (21.26)
40–49	1714 (24.96)	8570 (24.96)
50–59	1496 (21.79)	7480 (21.79)
60–69	719 (10.47)	3595 (10.47)
≥70	528 (7.69)	2640 (7.69)
Sex		
Male	4311 (62.79)	21,555 (62.79)
Female	2555 (37.21)	12,775 (37.21)
Diabetes		
Yes	1195 (17.40)	5975 (17.40)
No	5671 (82.60)	28,355 (82.60)
Hypertension		
Yes	2070 (30.15)	10,350 (30.15)
No	4796 (69.85)	23,980 (69.85)

*OSA* obstructive sleep apnea

### Events of chronic kidney disease

The primary end point was newly diagnosed CKD during the study period. The CKD diagnosis included 582 (chronic glomerulonephritis), 583 (nephritis and nephropathy not specified as acute or chronic), and 585 (chronic kidney disease) as previous reported on AJKD, and the nephritic syndrome was excluded due to the urine protein loss could not be confirmed from the database system. Newly diagnosed cases of CKD were identified as those who visited the outpatient care clinic or were hospitalized with a diagnosis of CKD (ICD-9-CM 582, 583, 585). However, to eliminate enrollment bias, we excluded the CKD episodes that occurred during the first year of follow up in this cohort study.

### Statistical analyses

For this analysis, follow-up time began on the index date of OSA and ended at the time of the CKD diagnosis, when the patient quit insurance, or on January 31, 2011, whichever came first. The descriptive results were displayed by the number of baseline characteristics among the OSA and non-OSA cohorts. The incidence of CKD was calculated based on person-years of follow-up for 2000–2011. Person-years of follow-up were estimated for each individual from the index date until the date of the CKD diagnosis, when the patient quit insurance, or on January 31, 2011. Kaplan-Meier methods and the log-rank test were used to examine the cumulative probability of CKD development according to the presence of OSA. The Cox proportional hazards models were used to analyze the association of baseline OSA with the risk of CKD with or without adjustment for age, sex, diabetes, and hypertension at baseline. The proportional hazards assumption was not violated, and hazard ratios (HRs) with 95 % confidence intervals (CIs) were calculated. Two-tailed tests were used and *p* values <0.05 were considered significant. The statistical analyses were performed using SAS version 9.2 (SAS Institute Inc., Cary, NC, USA) and STATA version 14.0 (STATA Corp, College Station, TX, USA).

## Results

**Fig. 2:**
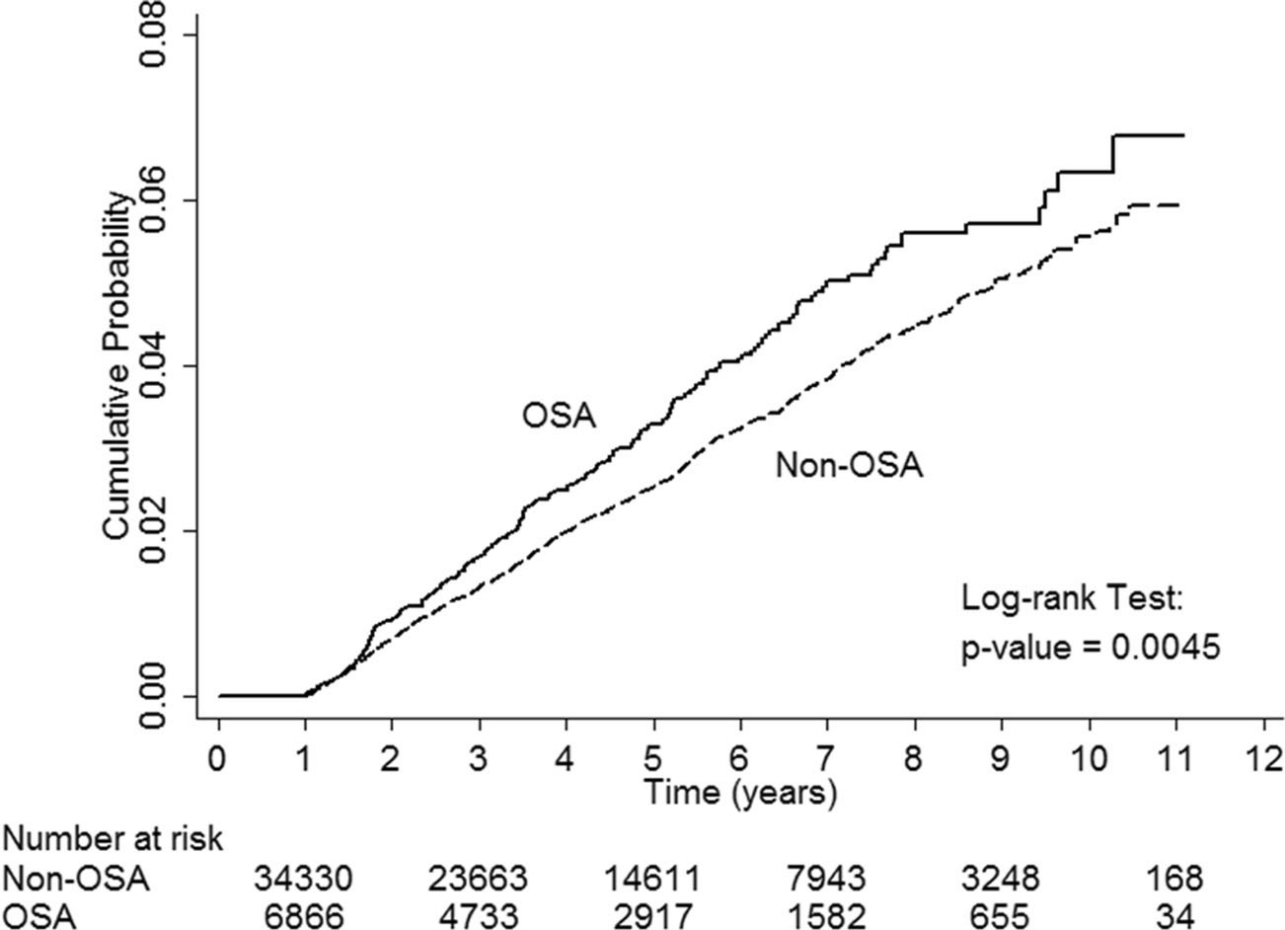
The cumulative probability of CKD development among the OSA and non-OSA cohort

The baseline demographics and clinical characteristics of the OSA and non-OSA cohorts are given in Table 1. There were no important differences between cohorts as expected due to the sample matching. We identified 6866 subjects with OSA who were followed up for a median of 4.4 years (total person-years = 33,402.5). In the non-OSA cohort, 34,330 subjects were matched to the OSA cohort and followed up for a median of 4.4 years (total person-years = 167,146.1). Of the 41,196 study subjects, 1082 reported CKD during the course of the study: 215 in the OSA cohort (median follow-up years, 3.2) and 867 in the non-OSA cohort (median follow-up years, 3.4). Patients in the OSA cohort developed CKD 2.5 months earlier than those in the non-OSA cohort did. The crude CKD event rate was higher for patients with OSA (23.3 outcomes per 1000 person-years) than for those without OSA (19.6 outcomes per 1000 person-years). With the Cox proportional hazards model, estimated HR (95 % CI) for risk of CKD with OSA versus non-OSA was 1.24 (1.07–1.44; *p* = 0.0045) (Table 2). After adjustment for age, sex, hypertension, and diabetes, the association between OSA and first CKD event remained significant. After excluding the confounding factors of CKD, hypertension, and diabetes, the risk of incident CKD persisted in the OSA cohort (HR, 1.37; 95 % CI, 1.05–1.77; *p* = 0.019) (Table 3). Figure 2 shows the cumulative probability of CKD development by OSA presence. The OSA cohort had higher cumulative incidence of CKD. The difference was statistically significant according to the log-rank test in comparison with the non-OSA cohort (*p* = 0.0045).

**Table 2:** Hazard ratio and 95 % confidence interval of incident CKD associated with OSA

	No. of cohort	No. of incident CKD	Incidence (per 1000 person-years)	Hazard ratio (95 % CI)	*p* value
OSA	6866	215	23.3	1.24 (1.07, 1.44)	0.0045
Non-OSA	34,330	867	19.6	Reference	

*OSA* obstructive sleep apnea, *CKD* chronic kidney disease

**Table 3:** Hazard ratios of CKD for OSA compared with non-OSA by cohort without diabetes or hypertension, age, and gender

	No. of cohort	No. of incident CKD	Incidence (per 1000 person-years)	Hazard ratio (95 % CI)	*p* value
Cohort without diabetes or hypertension					
OSA	4319	72	12.8	1.37 (1.05, 1.77)	0.019
Non-OSA	21,595	264	9.6		
Male					
OSA	4311	124	21.7	1.14 (0.94, 1.39)	0.19
Non-OSA	21,555	542	19.8		
Female					
OSA	2555	91	26.1	1.41 (1.12, 1.78)	0.0036
Non-OSA	12,755	325	19.3		
Aged 18–39 years					
OSA	2481	39	9.0	1.52 (1.01, 2.28)	0.046
Non-OSA	12,405	102	6.3		
Aged ≥40 years					
OSA	4457	185	31.3	1.21 (1.03, 1.42)	0.022
Non-OSA	22,285	768	27.0		

*OSA* obstructive sleep apnea, *CKD* chronic kidney disease

### Subgroup analyses

Table 3 also shows the relationship between OSA and the incidence of CKD in patients aged 18–39 years and ≥40 years as well as in men and women separately. An increase in the HR of the incidence of CKD due to OSA was observed in persons ≥40 years of age (HR, 1.21; 95 % CI, 1.03–1.42; *p* = 0.022) and borderline significant in persons 18–39 years of age (HR, 1.52; 95 % CI, 1.01–2.28; *p* = 0.045). Similarly, the risk of developing CKD was higher for women with OSA than for those without it (HR, 1.41; 95 % CI, 1.12–1.79; *p* = 0.0039). However, the risk of developing CKD among men with OSA was not significant.

## Discussion

Our group is the first to report a correlation between CKD and OSA severity in 40 OSA patients without hypertension and diabetes [15]. Later, Y et al. reported the association with a worse estimated glomerular filtration rate in 100 patients with CKD stage 3–4 and OSA [24, 25]. However, all these are small prevalence studies. Previous large longitudinal OSA cohort studies such as the Sleep Heart Health Study (SHHS) and Wisconsin Sleep Cohort Study (WSS) reported the associated adverse outcomes of hypertension, stroke, and mortality but not CKD outcomes [3, 4, 26]. Lee reported the predisposing association between OSA and CKD recently using the same Taiwan’s medical insurance database. However, due to statistic limitation, the selection bias about hypertension and diabetes cannot be clarified in their follow-up model. Also, the importance at young adult is difficult to evaluate due to the age selection (over 30 years old) and excluded from their study population. Our study from Taiwan’s one million medical insurance databases is the first large population-based cohort study to use a prospective statistical model to identify the increasing 1.37-fold incidence of CKD risk in pure OSA patients in Taiwan after excluding hypertension and diabetes. In addition to a higher risk of developing CKD, the OSA cohort developed CKD 2.5 months earlier than the non-OSA cohort did.

Previous studies have mounting evidences of OSA accelerating diabetes nephropathy [19, 27], and a small study and case report showed that CPAP treatment may improve proteinuria in diabetes patients [28, 29]. There are many factors including sleep cycle and the renin-angiotensin-aldosterone system [30, 31], intermittent hypoxia-induced endothelial dysfunction and artery stiffness [32–34], and renal tubular dysfunction-related nocturnal natriuresis [29, 35, 36] that may impact kidney function from OSA. In recent reviews, Dr Adeseun emphasizes intermittent hypoxia and sleep fragmentation-related kidney injury mechanism and possible more direct insults from intermittent hypoxia to CKD [37]. Dr Hanly reported more evidences suggest that OSA having direct impact on CKD especially from direct effect of intrarenal hypoxia and activation of the systemic and renal renin-angiotensin system but need further studies [38]. From the finding of our study, the evidence is obvious that OSA patients have impact on the deterioration of kidney function in a 12-year follow up. It is important to emphasize regularly checking the renal function and even the early renal injury markers in OSA patients, especially on the patients coexisting with other risk factors such as diabetes or hypertension. It is also important to emphasize the treatment of OSA such as CPAP therapy on the view of preventing the CKD.

Second, a difference in the outcomes between the sexes was noted by the SHHS and WSS, with a higher risk in men for stroke and hypertension, but all studies enrolled only patients >40 years with OSA. We found that the incidence of CKD was persistently high with a 1.5-fold risk in the OSA group aged 18–39 years, which has not been reported previously and needs further verification and evaluation. Finally, although the same trend was seen, the risk of CKD was statistically confirmed in women only. This interesting finding may be impacted by other inaccessible factors from this study, such as Chinese herb drug usage including aristolochic acid, which requires further verification [39].

However, there are limitations relating to the population-based ICD-9 code-recording database itself and others [19, 22]. First, environmental and risk factors such as aging, smoking, obesity, and metabolic syndrome could not be identified from the database and adjusted for in the analysis.

Second, the exact detailed laboratory data such as polysomnography information and proteinuria severity were inaccessible. Therefore, this study reported results from patients with OSA based on clinical diagnosis alone. This may underestimate the effects of severe OSA on CKD development. A prospective OSA cohort study is needed in the future to clarify the relationship between OSA severity and CKD.

In conclusion, this longitudinal population-based cohort study using a statistically prospective model provides the first evidence that patients with pure OSA without hypertension or diabetes are at higher risk of developing CKD over the next 3 years and nearly 2.5 months earlier than the non-OSA cohort, particularly women.
